# Automated recognition of cell phenotypes in histology images based on membrane- and nuclei-targeting biomarkers

**DOI:** 10.1186/1471-2342-7-7

**Published:** 2007-09-06

**Authors:** Bilge Karaçalı, Alexandra P Vamvakidou, Aydın Tözeren

**Affiliations:** 1 Center for Integrated Bioinformatics, School of Biomedical Engineering, Science and Health Systems Drexel University 3141 Chestnut Street Philadelphia PA 19104 USA

## Abstract

**Background:**

Three-dimensional *in vitro *culture of cancer cells are used to predict the effects of prospective anti-cancer drugs *in vivo*. In this study, we present an automated image analysis protocol for detailed morphological protein marker profiling of tumoroid cross section images.

**Methods:**

Histologic cross sections of breast tumoroids developed in co-culture suspensions of breast cancer cell lines, stained for E-cadherin and progesterone receptor, were digitized and pixels in these images were classified into five categories using *k*-means clustering. Automated segmentation was used to identify image regions composed of cells expressing a given biomarker. Synthesized images were created to check the accuracy of the image processing system.

**Results:**

Accuracy of automated segmentation was over 95% in identifying regions of interest in synthesized images. Image analysis of adjacent histology slides stained, respectively, for Ecad and PR, accurately predicted regions of different cell phenotypes. Image analysis of tumoroid cross sections from different tumoroids obtained under the same co-culture conditions indicated the variation of cellular composition from one tumoroid to another. Variations in the compositions of cross sections obtained from the same tumoroid were established by parallel analysis of Ecad and PR-stained cross section images.

**Conclusion:**

Proposed image analysis methods offer standardized high throughput profiling of molecular anatomy of tumoroids based on both membrane and nuclei markers that is suitable to rapid large scale investigations of anti-cancer compounds for drug development.

## Background

Fast, repeatable, and reliable methods are needed for evaluating the efficacy of prospective drugs in cancer research. Cell lines derived from cancer tissues are used extensively to model *in vivo *drug response as they can be transferred, reproduced, and analyzed in standardized assays [1,2]. Effects of therapeutic compounds have been studied widely on cell lines isolated from breast, skin, colon, prostate, lung, brain, and the bone marrow [3-9]. A comprehensive database of several human cancer cell lines' chemosensitivity to select anticancer drugs is presented by Dan et al[10]. The limitations of two-dimensional assays of cancer cell cultures in representing in vivo tissue conditions may be due to the lack of cell to cell and cell to extracellular matrix interactions [11]. Three dimensional cell cultures promote cell to cell interaction in a more realistic geometry [12]. Cancer cells grown in tumoroids interact with one another as well as with the extracellular matrix that they produce [13]. While the tumoroids grown *in vitro *lack the actual tissue micro environment known to affect the tumorigenic properties of cell lines [11,12,14], the high cost and low repeatability of *in vivo *tumor models in immune deficient host systems prevent standardized large scale and high throughput analyses. Therefore, *in vitro *tumoroids developed using cancer cell lines remain the primary models for standardized high throughput studies of cancer [15].

The potential benefits of automated image cytology are widely recognized for rapid and standardized assessment of biomarker status [16]. Comparisons of manual and automated methods in assessing biomarker expression showed a high correlation, establishing automated image analysis methods as effective and reliable alternatives to painstaking manual microscopic examinations [17,18]. Computational image analysis algorithms proposed in the literature for quantitative evaluation of histological tissue cross sections can be grouped under two categories based on the scale at which they characterize tissue anatomy [19,20]. The first group focuses on the appearance of cell nuclei [21-25], while the second group studies the appearance of cell clusters in terms of the spatial arrangement of cell nuclei as well as the texture characteristics of the tissue [26-29]. The expressions of nuclei-bound biomarkers were analyzed by detecting the nuclei in digitized histological cross-section images, and then classifying the positively- and negatively-stained nuclei within the field of view [30-33]. Lacking the well-defined spatial locus of the nucleus, membrane-bound biomarkers were assessed through densitometric analysis across larger tissue structures [34,35]. A fractal-based texture analysis method was proposed to identify positively and negatively stained tissue distributions in histology slides [36]. A software platform capable of combining different computational modules for processing histology images has been developed to enable designing custom image analysis pipelines with separate detection layers for nuclei and cell membranes in high-resolution histology images [37].

Open access systems operated by internet web servers, such as EAMUS™, have recently been developed for extracting quantitative parameters from immunohistochemically stained tissue slides [38,39] and tissue microarrays [40]. Several commercial software packages have also been developed for cytometric analysis of histological slides and tissue microarrays such as the Tissue Microarray Analysis Software (TMAx) by Beecher Instruments (Beecher Instruments, Inc., 686 Progress Way, Sun Prairie, WI 53590, USA), the Extended Slide Wizard by Tripath Imaging (Tripath Imaging, Inc., 780 Plantation Drive, Burlington, NC 27215, USA), the Discovery Image Analyser by Becton-Dickinson (Becton-Dickinson Biosciences, Postbus 757, 2400 AT Alphen aan den Rijn, The Netherlands), the TissueQuest software by TissueGnostics (TissueGnostics GmbH, Taborstraße 10/2/8, 1020 Vienna, Austria FN 234341 w) and the Automated Cellular Imaging System (ACIS) by Clarient (Clarient, Inc., 31 Columbia, Aliso Viejo, CA 92656, USA), along with the Stanford Tissue Microarray Analysis Software: CaseXplorer [41]. Among these, ACIS in particular has been used extensively, in the analysis of immunohistochemically stained lung cancer specimens for p53, ki-67, and p120 status [42] as well as HER-2/neu expression in breast cancer by both immunohistochemical staining and fluorescence in-situ hybridization [43-45]. However, a comprehensive methodology for automated analysis of histological sections of heterogeneous tumoroids stained for a variety of molecular markers, from construction of whole cross section images using overlapping snapshots to molecular profiling of DNA spots, has not been formalized. The differences in the microenvironment of the tumoroids to that of the actual tissue results in differences in the cross section images of tumoroids, and the computational algorithms used in studying histology slides obtained from surgically resected or biopsied specimens have not been validated on tumoroid cross section images. A typical example to these differences is a drastically increased number of overlapping nuclei due to much larger nuclear area [46].

We have studied the anatomy of co-cultures of poorly invasive and highly invasive breast cancer cell lines using digitized cross section images immunohistochemically stained for E-cadherin (Ecad) and progesterone receptor (PR) by automated image analysis methods. Positively and negatively stained regions of cross section images were delineated using image segmentation algorithms based on pixel color. The DNA spots were identified in positively and negatively stained image regions. Our results showed that the Ecad+/PR+ and Ecad-/PR- cell lines exhibited strong homophilic binding. This preference was more pronounced in invasive cells which produced several Ecad/PR deficient tumoroids. All image analysis algorithms were validated on synthetic images for segmentation accuracy and DNA spot profiling performance. These results indicated that the tumoroids developed using mixture cell suspensions were anatomically heterogeneous, and the automated image analysis methods developed in this study enabled rapid and accurate morphological phenotyping of such tumoroids using immunohistochemical staining for both membrane and nuclear targets.

## Methods

### Image Data

The image data used in this study were obtained by digitizing histological cross sections of composite breast tumoroids described in our previous publication [47]. Drexel University Institutional Review Board reviewed our research involving the histology slides of in vitro tumoroids composed of cultured cancer cells and determined that it was in compliance with Drexel university research policy involving biological samples. Briefly, cultured human breast cancer cells of highly invasive (MDAMB231) and poorly invasive (MCF7, and ZR751) phenotypes were co-cultured at respective concentrations of 25% and 75% in a rotating wall vessel bioreactor to form a large number of tumoroids. These large cell aggregates were harvested after 8 days of culture in a rotating wall vessel (RWV) bioreactor (10 mL disposable High Aspect Ratio Vessel, HARV, Synthecon Inc., Houston TX). Poorly invasive cell lines used in co-culture were estrogen receptor (ER), progesterone receptor (PR), and E-cadherin (Ecad) positive [48-51] whereas the highly invasive MDAMB231 breast cancer cells were ER, PR and Ecad negative [52]. Tumoroids were processed for routine histology and immunohistochemistryby fixation in 10% neutral buffered formalin (Formalde-Fresh, Fisher), dehydrated by a series of alcohol baths and embedded in paraffin. Embedded tumoroids were sectioned at 10 *μm *intervals. Serial sections were prepared and were stained with hematoxylin and eosin to identify RNS regions in blue. In addition, ten of these slides were PAP-stainedfor Ecad (mouse anti-E-cadherin, clone 4A2C7, Zymed Laboratories Inc., 561 Eccles Avenue, South San Francisco, CA, 94080) and ten others for PR (DAKO monoclonal mouse anti-human progesterone receptor, clone PgR 636, DAKO Corporation, 6392 Via Real, Carpinteria, CA, 93013). Ecad-staining exemplified membrane-targeting biomarkers, and PR-staining exemplified nuclei/cytoplasm-targeting biomarkers.

**Figure 1:**
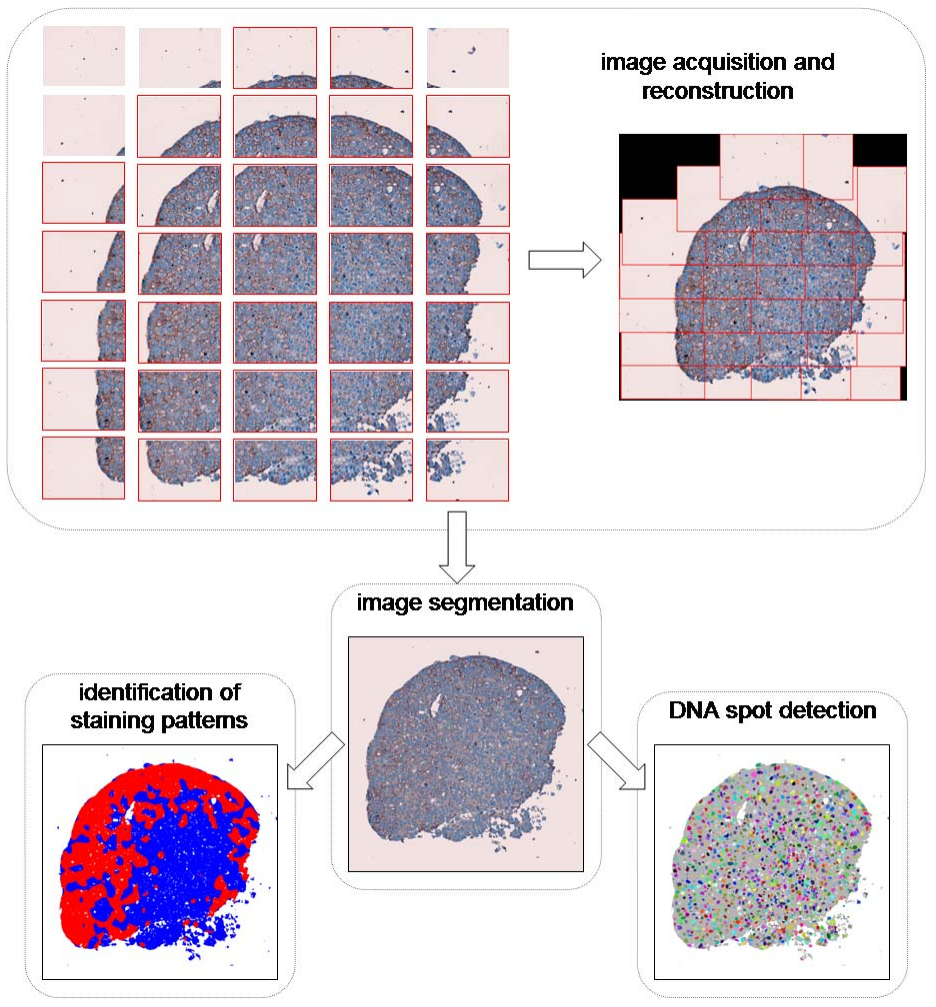
Image processing pipeline used to analyze immunohistochemically stained tumoroid cross section images. After the tumoroid cross sections were digitized at 40 ×, different tissue components were identified using unsupervised clustering and classification. Using the spatial distribution of pixels associated with positively and negatively stained cells, the staining patterns were identified. At the same time, the spatial distributions of DNA-rich pixels were analyzed and individual DNA spots were identified.

Digitized images of histology sections of breast tumoroids obtained under co-culture conditions were collected at H40 magnification scale using Coolscope VS Digital Microscope (Nikon, Kanagawa, Japan) and the dedicated Coolscope VS Suite (Bacus, IL) imaging software. Image collection involved consecutive overlapping snapshots at a color depth of 24 bits using the graphical user interface of the Coolscope VS Suite. These snapshots were later merged in software to produce a single image containing individual tumoroid cross sections. The pixel sizes of the resulting images were 0.17 μm H 0.17 μm (Figure 1).

Prior to processing, the color compositions of all image pixels were converted from the native RGB representation to the CIEL*a*b* representation [53]. The CIEL*a*b* color space is characterized by the luminance L* along with the chromaticity indices a* and b*. The details of this color conversion are provided in the Appendix. One advantage of using the CIEL*a*b* color representation instead of the RGB representation native to the image acquisition system is the linearization of the color differences: The CIEL*a*b* color space is designed to capture the nonlinear perception of the human visual system, so that the Euclidean distances between colors in the CIEL*a*b* space mimic closely the differences perceived by an average human eye [53]. The CIEL*a*b* space provides a device independent color representation by factoring in the white point of the image acquisition device. This enables standardized processing of images obtained using different imaging systems.

### Segmentation of cell clusters based on membrane targeting biomarkers

Pixel classes in Ecad-stained tumoroid cross section images were determined using *k*-means unsupervised clustering on a reference image [54]. The reference image was selected to exhibit all the regions of interest for studying the tumoroid cross-section images in our dataset in abundance, and therefore, to present the most complete and statistically accurate description of the data for training the classifiers. The segmentations achieved on the reference image for *k *= 2, 3, 4, 5, and 6 classes were evaluated visually for their ability to distinguish different tumoroid cross section image constituents. The segmentation obtained using *k *= 5 delineated the following pixel classes presented in the order of light to dark: *E1*: bright pixels in regions void of tissues and cells; *E2*: pixels that belonged to cell cytoplasm; *E3*: pixels that belonged to chromatin rich regions such as the cross sections of cell nuclei; *E4*: pixels that belong to cell cytoplasm stained by diffusing Ecad staining on the cell membrane; and *E5*: the pixels that were on the membranes of the Ecad+ cells and their immediate vicinity. The cluster centers determined by the 5-class *k*-means clustering for the reference image were used in the segmentation by nearest neighbor classification of the images of Ecad-stained histology slides used in this study. Note that this algorithm was designed to identify pixel clusters that exhibited statistically conspicuous color separation, and not to quantify the actual intensity of staining.

Classification of pixels in images decorated by protein markers for Ecad into five categories was a first step in the automated delineation of the regions of the histology images composed of cells expressing the protein marker. In Ecad-stained cross section images, these regions typically contained pixels from four of the five categories described above (*E2 *to *E5*). Image regions that consisted of Ecad expressing cells were determined via automated classification of individual image pixels into belonging to either Ecad expressing or Ecad negative categories by thresholding of a weighted ratio of pixels that are in *E4 *and *E5 *classes in the immediate neighborhood of the pixel. The size of this neighborhood was determined by the standard Gaussian smoothing with standard deviation *τ *= 6.30 μm. This region covers the diameters of two typical cells within an interval ± 2*τ *in order to obtain congruent region delineations. The optimal threshold separating image regions containing Ecad positive and Ecad negative cells was determined by carrying out a *k*-means unsupervised clustering (*k *= 2) on the set of ratios from the reference image after the set of staining ratios have been normalized to optimally span the unit interval. The threshold on the staining ratios was determined to be 0.1875, and was used to determine the positively stained regions in all Ecad-stained tumoroid cross section images.

### Segmentation of cell clusters based on nuclei/cytoplasm targeting biomarkers

Classification of the pixels in PR-stained tumoroid cross section images followed the same strategy as above. First pixel classes in a PR-stained reference image were determined via *k*-means clustering for *k *= 2, 3, 4, 5, and 6. The segmentation with 5 classes was determined visually to best capture the pixel types present in the cross section image. The pixels in PR decorated images were classified into the following categories: *P1*: pixels that belonged to tissue void regions; *P2*: pixels that fell on the cytoplasm of PR negative cells, *P3*: pixels that fell on the cytoplasm of PR positive cells; *P4*: pixels that corresponded to the chromatin containing regions in PR negative cells; and *P5*: pixels that belonged to chromatin-rich regions in PR positive cells. The cluster centers of these five classes were determined by the *k*-means unsupervised clustering. Then, the images of the PR-stained tumoroid cross sections used in the study were segmented by nearest neighbor classification using these cluster centers obtained for the reference dataset.

The positively stained regions in PR-stained tumoroid cross section images were determined using a similar method as for Ecad-stained cross section images. An optimal threshold of 0.185 was determined by *k*-means unsupervised clustering with *k *= 2 on the positive staining ratios from the reference PR-stained tumoroid cross section image after normalization (classes *P3 *and *P5*). This threshold was later used to identify the positively stained regions in all the remaining PR-stained cross section images.

### Accuracy of segmentation: testing with synthetic images

In order to elucidate the operating characteristics of the image analysis algorithms used in the manuscript in a controlled setting, we have carried out validation experiments using synthesized Ecad and PR stained cross section images. First, we have randomly generated a three dimensional tissue block in a 128 μm × 128 μm × 64 μm volume. To this end, we have randomly placed a total of 489 seeds for individual cells, and partitioned the volume among these seeds using a nearest neighbor rule. This number corresponds to the number of idealized 16 μm-wide spherical cells required to fill this volume. We have then eliminated the cells that reside at the 4 sides of the tissue block, and randomly placed ellipsoid nuclei inside the remaining cells. A total of 9 horizontal cross sections were obtained from this tissue block at 4 μm intervals around the midsection, and the membranes, cytoplasms, and the nuclei of positively and negatively stained cells were marked. The staining status of each cell was also assigned randomly.

Ecad and PR stained cross section images were generated by randomly assigning colors to tissue map pixels from among those observed in the reference tumoroid cross section images in the corresponding pixel classes. The color images were also smoothened in order to emulate the effects of the point spread function of image acquisition. A total of 45 synthetic Ecad and Pr stained image pairs were generated from 5 independently synthesized tissue blocks, and the performances of the computational methods were assessed in terms of tissue segmentation as well as molecular and morphological profiling of DNA spots.

### Accuracy of segmentation using adjacent histology slides decorated with membrane-targeting and nuclei-targeting biomarkers

Segmentation accuracy on actual images was assessed using the staining patterns obtained for adjacent histology slides that were stained for Ecad and PR. Since the cells in the composite tumoroids were either Ecad+ and PR+ or Ecad- and PR-, the agreement between the staining patterns in Ecad-stained and PR-stained cross section images provided additional validation for the tissue segmentation algorithms.

Two measures of aerial concentrations of cell phenotypes in a given tumoroid cross section image were used. The first measure was obtained by the percentage of positively and negatively stained regions across the whole cross section image. In addition, the detected DNA spots were classified as positively-stained or negatively stained based on the whether their centers of mass were in positively or negatively stained regions. The percentage of positively and negatively stained DNA spots provided the second measure of cell phenotype concentration in tumoroid cross section images. These two measures of cell concentration were then compared and contrasted between Ecad-stained and PR-stained cross section images.

### Nuclei DNA spot detection and testing validity using synthetic images

The DNA-rich pixels in Ecad-stained tumoroid cross section images are identified by image segmentation as the *E3 *class and those in PR-stained cross section images are identified as *P4 *and *P5 *classes. The indicator functions of these classes across respective images define the binary maps of DNA-rich pixels. First, we have refined these initial DNA-rich pixel maps by subsequent opening and closing operations of mathematical morphology using circular structuring elements of radii 0.85 μm and 0.52 μm respectively. This process eliminated singleton DNA-rich pixels and enhanced the spatial definitions of the DNA spots. The remaining DNA blobs were further analyzed using a watershed transform and individual DNA spots were identified [55]. This procedure identified all spots of DNA-associated pixels regardless of whether they belong to nucleus or mitochondria. A *k*-means unsupervised clustering with *k *= 2 was used to determine the optimal area threshold that separates the small DNA spots that are most likely to be associated with mitochondria than nuclei. DNA spots identified in Ecad-stained tumoroid cross section images were deemed to be nuclei cross sections if their area *A *was greater than 41.56 μm^2^. In PR-stained cross section images the threshold value for the DNA spot area *A *was 36.2 μm^2^.

We have identified a series of morphological parameters to characterize the shapes of the DNA spots identified in the tumoroid cross section images. These parameters included the cross sectional area of a DNA spot (*A*) and the eccentricity ratio defined as E=1−βα
 MathType@MTEF@5@5@+=feaafiart1ev1aaatCvAUfKttLearuWrP9MDH5MBPbIqV92AaeXatLxBI9gBaebbnrfifHhDYfgasaacH8akY=wiFfYdH8Gipec8Eeeu0xXdbba9frFj0=OqFfea0dXdd9vqai=hGuQ8kuc9pgc9s8qqaq=dirpe0xb9q8qiLsFr0=vr0=vr0dc8meaabaqaciaacaGaaeqabaqabeGadaaakeaacqWGfbqrcqGH9aqpcqaIXaqmcqGHsisldaWcaaqaaGGaciab=j7aIbqaaiab=f7aHbaaaaa@33F4@ where *α *and *β *denote the major and minor axis of the best fitting ellipsoid to the DNA spot. In addition, we have computed the mean inter-spot distance (*D*) for each DNA spot by taking the average of the Euclidean distances from its center of mass to those of the nearest 10 DNA spots. We have also determined whether each identified DNA spot resides in a positively stained region or a negatively stained region of a tumoroid cross section image based on the location of its center of mass and the previously obtained cross sectional staining patterns. Note that since the cross sectional areas of the retained DNA spots were much larger than the structural elements used for morphological filtering, the subsequent effects on the shape parameters were insignificant.

We have assessed the accuracy of the DNA spot detection and morphological profiling algorithms on the synthetic images generated previously. Specifically, we have used these algorithms to identify the DNA spots in the synthetic images and measure their morphological parameters. We have then computed the DNA spot detection rates and the error statistics on morphological parameters by comparing the measured spot areas, eccentricities, and average inter-spot distances to the actual values averaged over 10 synthetic images.

## Results

**Figure 2:**
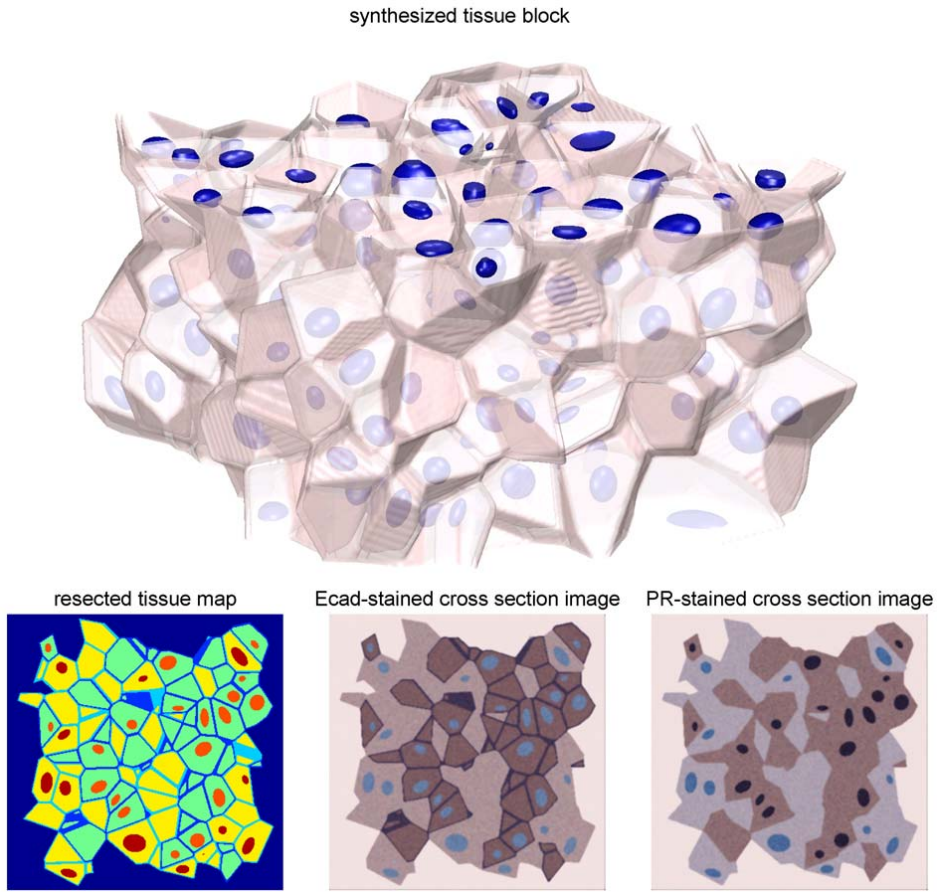
Sample synthetic images used to validate the computational image analysis algorithms used in the manuscript. First, a three-dimensional tissue block was generated randomly within a 128 μm × 128 μm × 64 m volume. Random tissue maps were obtained as 9 horizontal cross sections of this tissue block at 4 μm intervals near the vertical center, where the membranes, cytoplasms, and the nuclei of Ecad+/PR+ and Ecad-/PR- cells were marked with different labels. The associated Ecad-stained and PR-stained cross section images were generated by assigning colors from among those observed in the reference tumoroid cross section images followed by smoothing (bottom row). Overall, 5 such tissue blocks were generated, producing a total of 45 random tissue maps and respectively stained synthetic images. Image acquisition was modeled at 40×.

The automated methods for image processing were first tested on synthetic images computationally simulating the biomarker decorated images of the tumoroid cross sections (Figure 2). The accuracy of tissue segmentation was 95.82% and 98.31% on average over synthetic images mimicking Ecad and PR stained tissue cross section images respectively (Tables 1 and 2). Morphological profiling of DNA spots in terms of cross sectional area, eccentricity, and mean inter-spot distances over synthesized tumoroid cross-section images adequately captured the true parameters of the DNA spots (Table 3). DNA spot detection rates were over 93% both for Ecad-stained and PR-stained synthetic cross-section images. The spots that were missed were less than 5 μm^2 ^in cross sectional area. The average absolute errors in positively stained region areas were less than 6% of the average area of synthesized cross section images. The average error in DNA spot areas in the PR-stained images was higher than that in Ecad-stained images, possibly due to a two-class representation of DNA-rich pixels in PR-stained images. Conversely, the error in identifying the staining of the DNA spots was lower in PR-stained images than that in Ecad-stained images, as the PR stain directly targets the DNA-rich image content. The errors in spot eccentricities and inter-spot distances were comparable between the synthetic images representing Ecad and PR-stained tumoroid cross section images.

**Table 1:** Normalized confusion matrix of automated image segmentation on the synthesized Ecad-stained cross section images. The overall prediction accuracy measured by the sum of diagonal elements was 0.9582.

True class	Predicted class					
	
	E1	E2	E3	E4	E5	total
E1	0.3457	0.0039	0.0000	0.0001	0.0000	0.3498
E2	0.0014	0.2832	0.0007	0.0062	0.0004	0.2919
E3	0.0000	0.0008	0.0426	0.0008	0.0000	0.0441
E4	0.0000	0.0000	0.0009	0.2270	0.0122	0.2400
E5	0.0001	0.0024	0.0001	0.0120	0.0596	0.0742
Total	0.3472	0.2902	0.0443	0.2461	0.0722	1.0000

**Table 2:** Normalized confusion matrix of automated image segmentation on the synthesized PR-stained cross section images. The overall prediction accuracy measured by the sum of diagonal elements was 0.9831.

True class	Predicted class					
	
	P1	P2	P3	P4	P5	total
P1	0.3460	0.0038	0.0000	0.0000	0.0000	0.3498
P2	0.0013	0.2886	0.0016	0.0003	0.0000	0.2919
P3	0.0003	0.0066	0.3068	0.0000	0.0005	0.3142
P4	0.0000	0.0012	0.0000	0.0200	0.0000	0.0212
P5	0.0000	0.0000	0.0012	0.0000	0.0217	0.0230
Total	0.3476	0.3002	0.3096	0.0203	0.0222	1.0000

**Table 3:** Error statistics of the image analysis methods used for determining the staining patterns and detecting DNA spots on the synthesized cross section images. The statistics were averaged over 45 randomly generated cross section images.

	Ecad-stained images	PR-stained images
True DNA spot detection rate	93.77%	93.07%
False DNA spot detection rate	0.00%	0.00%
Absolute error in positively stained area (μm^2^)	615.2412	537.0872
Absolute error in DNA spot area (*A*) (μm^2^)	0.4031	1.1964
Absolute error in DNA spot eccentricity (*E*)	0.0270	0.0244
Absolute error in inter-spot distances (*D*) (μm)	1.0314	1.1536
Error rate of staining prediction	8.38%	6.56%

**Figure 3:**
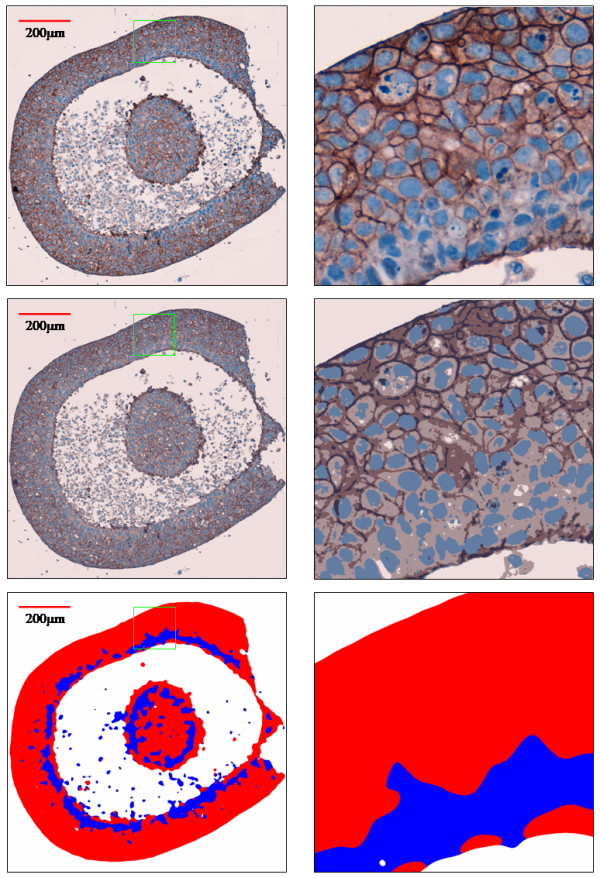
Processing of membrane-targeting Ecad stained tumoroid cross section images. The original image at 40× (top), the segmentation (middle), and the deduced staining patterns are shown (left column) along with marked high magnification boxes of width 156 μm (right column).

**Figure 4:**
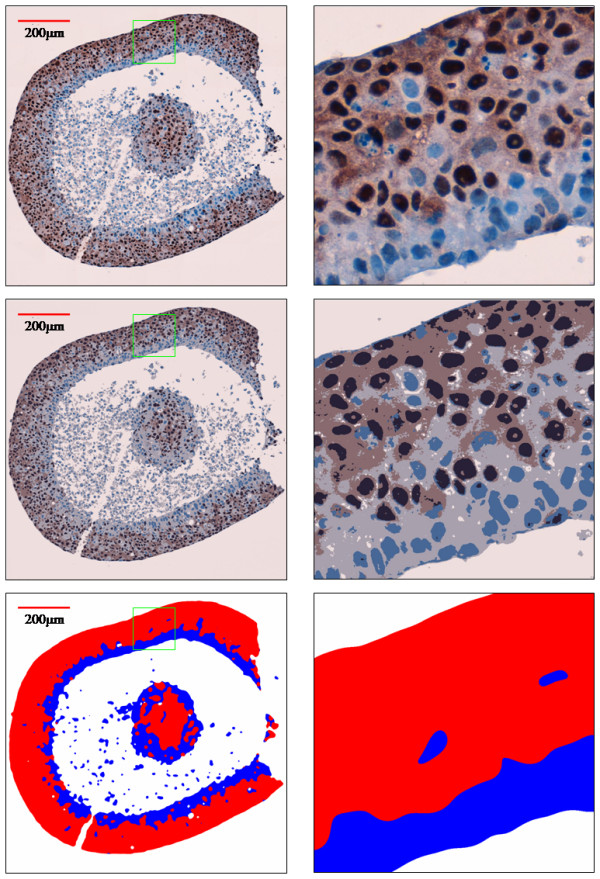
Processing of nuclei-targeting PR stained tumoroid cross section images. The original image at 40× (top), the segmentation (middle), and the deduced staining patterns are shown (left column) along with marked high magnification boxes of width 156 μm (right column).

Visual inspection of histology images of tumoroid sections decorated for Ecad or PR indicated that in many instances these cross sections cut across cells of highly invasive and poorly invasive phenotypes (Figures 3 and 4). The tumoroid shown in the figures is in the form of a cell-rich shell encompassing a largely cell free core. As the tumoroid grew with increasing durations of incubation, cell free core regions became apparent much more frequently, possibly as a result of insufficient diffusion of nutrients and oxygen across the thickness of the tumoroid. The tumoroid shells in these images ranged in thickness from 100–140 μm. The presence of small cell clusters of different phenotypes on the same cross section side by side can be observed clearly in Figures 3 and 4, indicating that cells belonging to different phenotypes are able to adhere to each other even in the absence of E-cadherin (homotypic cell-cell adhesion molecules). Similarly, the visual inspection of the same figure shows that PR staining is also efficient in identifying the microscopic boundaries of cell clusters belonging to different phenotypes. The automated segmentation procedure described in the methods section separates image regions occupied by highly invasive cells (blue) from those regions occupied by poorly invasive cells at a coarser scale. The visual comparison of top and bottom rows of Figures 3 and 4 indicate that automated segmentation correctly captures the presence of different cell phenotypes in a histology slide image globally, without the need for visualization at the physical dimensions of a living cell.

**Figure 5:**
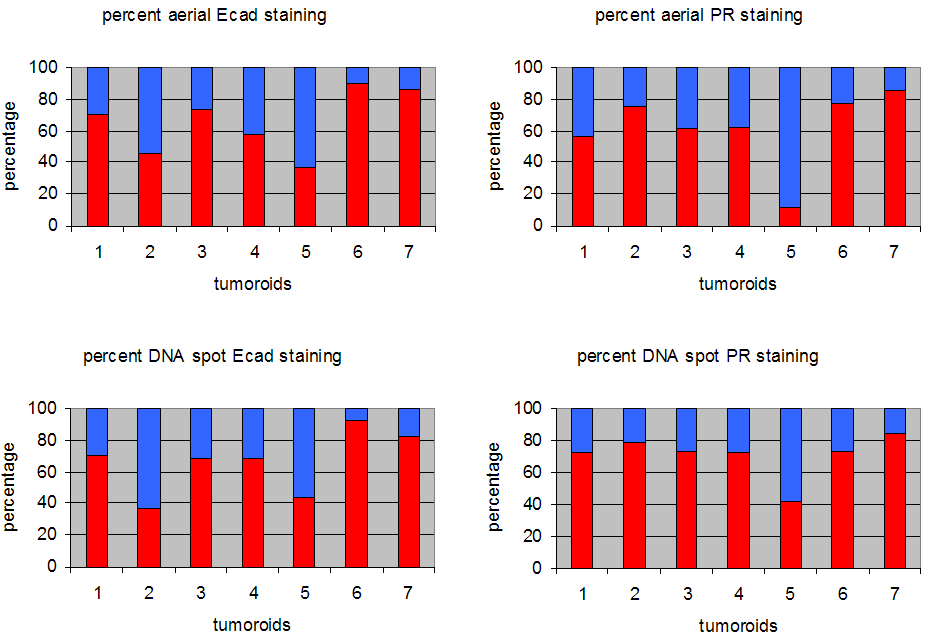
Aerial and DNA spot percentages of positively and negatively stained regions. The percentages based on areas and the number of DNA spots in positively and negatively stained regions were very similar between different cross sections of tumoroids. The positive staining percentages of PR and Ecad staining followed each other in general to within a difference of 12% even though differences as large as 30% were also observed. In the charts above, positive staining is shown in red and negative staining is shown in blue.

The automated image analysis showed that composite tumoroids that were developed using poorly invasive to highly invasive cells at a three-to-one ratio contained largely cells of poorly invasive phenotype (Figure 5). On the average, invasive cell phenotype occupied 34 % of the tumoroid cross section in images stained for Ecad and 39% of the cross section in images stained for PR. The composition of a tumoroid cross section varied from cross section to cross section for tumoroids developed under identical co-culture conditions as shown in Figure 5. Adjacent cross sections of the same tumoroid stained for PR and stained for Ecad, however, showed closer prediction of the highly invasive cell phenotype regions. These results indicate the cell phenotype composition of a composite tumoroid cannot be estimated accurately by computing the corresponding composition in the images of a few tumoroid cross sections. On the other hand, automated segmentation developed in this study allows for the creation of tissue arrays from composite tumoroid cross sections for high throughput studies on potential drugs.

## Discussion

As modern pathology explores the use of automated image processing systems for accurate diagnosis of cancer class, similar automated techniques are needed to analyze images of tissue microarrays containing images from hundreds of different tissues in a single array. Tissue arrays that correspond to adjacent slides of a composite block are stained for different biomarkers [56-59] and therefore it is necessary to identify the image regions that belong to cells expressing a particular biomarker. The computational image analysis algorithms used in this manuscript were validated over synthesized Ecad and PR stained cross section images. The results shown in Tables 1, 2, and 3 indicated high accuracy in tissue segmentation as well as molecular and morphological profiling of DNA spots in synthetic images. In comparison, measurements of DNA spot areas were more accurate in Ecad-stained cross section images, while DNA spot staining was determined more accurately in PR-stained cross section images. Overall, the performances of the image analysis methods were comparable on synthesized image data for both stains. While the synthesized cross-section images provide a simplified view of real histology imagery in terms of geometry and optical characteristics, these results illustrate the operating characteristics of the image processing algorithms in assessing the expressions of membrane- and nuclei-bound biomarkers.

Since the image analysis methods presented in this paper operate on a pixel-based approach in identifying the staining of cell nucleus or membrane, they can be easily generalized to analyze sub-cellular staining characteristics of tumoroid cross-section images as well. Estimation of cell boundaries would be required to assess cytoplasmic staining on a per cell basis [60]. In such instances, the image resolution stands the critical issue in recognizing positively stained pixels, since small pixel clusters may be lost through the course of mathematical morphological operations. Image resolution also underlines the distinction of the pixel-based strategy presented here from an object-based approach aimed at recognizing cellular structures in cross-section images first and then examining their staining properties [38]. Object recognition is inherently based on geometric detail, and requires resolution levels much finer than the typical size of the objects of interest. On the other hand, object-based strategies can achieve greater robustness to variations in tissue preparation and imaging conditions by invoking the geometric information in the process through model-based segmentation, such as resolving instances of partial staining and overlapping nuclei [61].

The present image processing study involving *in vitro *tumoroid cross sections showed that these tumoroids capture the heterogeneous composition of human breast tumors. Moreover, our automated image analysis is capable of identifying image regions correspond to cells expression different biomarkers. The two biomarkers that we tested our image analysis rest in different regions of a breast cell: Ecad is located on cell boundaries whereas PR is a protein that is located in the nucleus as well as in the cell cytoplasm. Stains for these biomarkers often diffused around the biomarker in the tumoroid slides. Our image segmentation took stain diffusion into consideration via separate pixel classes representing cytoplasmic expression of respective biomarkers, and was able to identify regions of interest even in the presence of significant biomarker diffusion.

## Conclusion

This article presents an automated analysis of breast tumoroid cross sections that are stained for cell nuclei and further decorated with nuclear and membrane bound biomarkers. Specifically we have developed imaging tools that differentiate various cell types in the image based on cell membrane bound and nuclear bound protein markers. Our automated image processing codes take into account the inadvertent diffusion of stains that occurs in many histology slides and differentiates different cell phenotypes on the image, allowing scientists to determine computationally the biological region of interest. The synthetic images generated in this article could be used as standard images in assessing the effectiveness of histopathology image segmentation tools in differentiatiating between cell phenotypes. The need for automated image analysis and classification of tumoroid histology will increase with further incorporation quantum dots [62,63] and other nanosystems into tissue microarray technology for keeping track of multiple biomarkers on the images of the same histology slide. The accuracy at which three-dimensional cell cultures emulate actual tumor tissue and *in vivo *tissue microenvironment can be improved further by co-culturing not only different cell lines but also cells of stromal origin [12]. Coupled with the automated image analysis methods as presented here for rapid profiling of the molecular anatomy of the resulting tumoroids, this offers a great potential for standardized high throughput evaluation of prospective drugs for anti-cancer performance.

## Competing interests

The author(s) declare that they have no competing interests.

## Authors' contributions

BK participated in the design of the study, digitized the tumoroid cross section images, carried out the analysis, and helped to draft the manuscript. AV developed the tumoroids from co-cultures of breast cancer cell lines and participated in the design of the study. AT conceived of the study, participated in its design and coordination, and helped to draft the manuscript. All authors read and approved the final manuscript.

## Pre-publication history

The pre-publication history for this paper can be accessed here:


